# Elusive boundaries: using an attribute framework to describe systems for population physical activity promotion

**DOI:** 10.1093/heapro/daae003

**Published:** 2024-02-02

**Authors:** Lori Baugh Littlejohns, Drona Rasali, Geoffrey McKee, Daniel Naiman, Guy Faulkner

**Affiliations:** BC Centre for Disease Control, Population and Public Health, 655 W 12th Avenue, Vancouver, BC V5Z 4R4, Canada; School of Kinesiology, University of British Columbia, 210-6081 University Boulevard, Vancouver, BC V6T 1Z1, Canada; School of Population and Public Health, University of British Columbia, 2206 E Mall, Vancouver, BC V6T 1Z3, Canada; School of Population and Public Health, University of British Columbia, 2206 E Mall, Vancouver, BC V6T 1Z3, Canada; Population and Public Health, BC Centre for Disease Control, 655 W 12th Avenue, Vancouver, BC V5Z 4R4, Canada; Healthy Schools and Physical Activity, BC Ministry of Health, Stn Prov Gov, PO Box 9646, Victoria, BC V8W 9P1, Canada; School of Kinesiology, University of British Columbia, 210-6081 University Boulevard, Vancouver, BC V6T 1Z1, Canada

**Keywords:** complex systems thinking, population physical activity promotion

## Abstract

The cost of physical inactivity is alarming, and calls for whole-of-system approaches to population physical activity promotion (PPAP) are increasing. One innovative approach to PPAP is to use a framework of interdependent attributes and associated dimensions of effective systems for chronic disease prevention. Describing system boundaries can be an elusive task, and this article reports on using an attribute framework as a first step in describing and then assessing and strengthening a provincial system for PPAP in British Columbia, Canada. Interviews were conducted with provincial stakeholders to gather perspectives regarding attributes of the system. Following this, two workshops were facilitated to document important stories about the current system for PPAP and link story themes with attributes. Results from interviews and workshops were summarized into key findings and a set of descriptive statements. One hundred and twenty-one statements provide depth, breadth and scope to descriptions of the system through the lens of an adapted framework including four attributes: (i) implementation of desired actions, (ii) resources, (iii) leadership and (iv) collaborative capacity. The attribute framework was a useful tool to guide a whole-of-system approach and turn elusive boundaries into rich descriptors of a provincial system for PPAP. Immediate implications for our research are to translate descriptive statements into variables, then assess the system through group model building and identify leverage points from a causal loop diagram to strengthen the system. Future application of this approach in other contexts, settings and health promotion and disease prevention topics is recommended.

**Contribution to Health Promotion**

- The cost of physical inactivity is alarming, and calls for whole-of-system approaches to population physical activity promotion are increasing.
- One innovative approach is applying a framework of interdependent attributes of effective systems for chronic disease prevention.
- An adapted framework includes the attributes of leadership, resources, implementation of desired actions and collaborative capacity.
- This framework was a valuable tool in defining often elusive boundaries to describe systems for health promotion.
- Implications for our research are to translate descriptions into variables to assess the system through group model building and identify leverage points from a causal loop diagram to strengthen the system.

## BACKGROUND

Globally, physical inactivity is the fourth leading risk factor for premature death (after high blood pressure, smoking and diabetes), and cost estimates of physical inactivity are estimated to be US$520 billion (2020–2030) (Santos *et al*., 2023). Elevating physical activity (PA) levels is a global challenge and the ‘largest economic cost is set to occur among high-income countries, which will account for 70% of health-care expenditure on treating illness associated with physical inactivity’ (World Health Organization, 2022). In British Columbia, Canada, where the authors live and work, the estimated direct and indirect costs of physical inactivity in relation to cardiovascular disease are calculated to approach CDN$700 million per year (Krueger *et al*., 2018).

The economic case for population physical activity promotion (PPAP) is well articulated, and the impacts of PPAP on improved health and quality of life (e.g. mental health, mobility) and possible mitigation of climate change (e.g. active transportation) are also clear (World Health Organization, 2018). PPAP is defined in this article as health promotion and chronic disease prevention efforts focused on planning, implementing and evaluating initiatives to improve PA levels at the population and system level. There are few areas in public health such as PPAP where cost-effectiveness and health impact are so convincing yet implementation of policies and practices remains low (Rutter *et al*., 2019; World Health Organization, 2022).

Increasing PA levels among a population is a particularly challenging and complex problem because of the multiple interacting factors that influence behaviour. There are examples of research that focus attention on identifying relationships among factors that influence PA behaviour and offer potential policy and practice interventions or actions (Holdsworth *et al*., 2017; Cavill *et al*., 2020; Guariguata *et al*., 2021; Waterlander *et al*., 2021). Here, interventions or actions refer to those that target PA behaviour (e.g. school-based PA policies and programmes). However, Hawe *et al*. (2009) suggest that in health promotion ‘the most significant aspect of the complexity possibly lies not in the intervention per se (multi faceted as it might be), but in the context or setting into which the intervention is introduced and with which the intervention interacts’. They further explain that complexity could be conceptualized more in terms of characteristics of the system rather than the interventions or actions. This article builds upon a complex system perspective as opposed to a focus on the complex problem of PA behaviour change per se.

Two recent World Health Organization reports describe the need for a focus on strengthening complex systems for PPAP. First, the *Global Action Plan on Physical Activity* (GAPPA) (World Health Organization, 2018) calls for creating active systems as one of four strategic objectives (including active societies, active environments and active people). Creating active systems include strengthening leadership (governance, policy frameworks and coherence), multisectoral partnerships (engagement and coordination), workforce capabilities, advocacy (multisectoral), information (surveillance and monitoring, research), resources (financing) and implementation of actions. Second, advocacy for system-level multisectoral collaboration and coordinating mechanisms to increase implementation of actions to address physical inactivity was a key recommendation in the *Global Status Report on Physical Activity 2022* (World Health Organization, 2022). There are a few examples in the literature that focus attention on complex systems for PPAP, but the field is in its infancy (Brennan *et al*., 2015; Bellew *et al*., 2020).

**Fig. 1:**
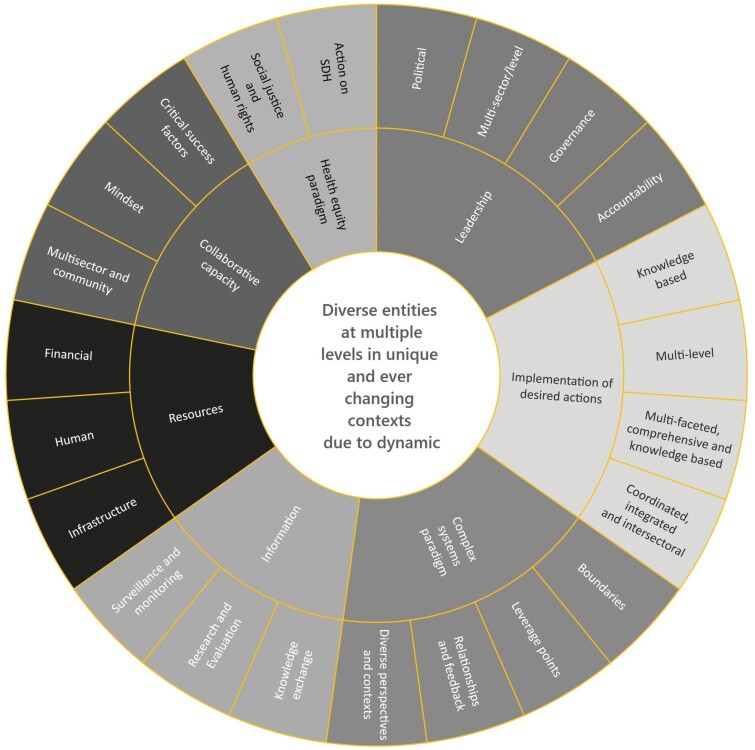
Attribute framework as reported by Baugh Littlejohns and Wilson (2019).

Stafford Beer (2002), a systems theorist from the UK, coined the phrase ‘the purpose of the system is what it does’; however, it is often difficult to describe what a system does in a comprehensive manner. To help systematically explore this gap, Baugh Littlejohns and Wilson (2019) developed a framework from a scoping review to identify key characteristics of effective systems for chronic disease prevention. A system was described in terms of ‘diverse entities at multiple levels in unique and ever changing contexts due to dynamic relationships among entities and actions’ (Baugh Littlejohns and Wilson, 2019). The framework in Figure 1 articulates seven different attributes (leadership, resources, implementation of desired actions, information, health equity paradigm, collaborative capacity and complex system paradigm) and 23 associated dimensions.

The framework is a group of interdependent attributes and dimensions and sets the stage for identifying what systems for chronic disease prevention do in each of these areas and the relationships among them. To our knowledge, only Bensberg *et al*. (2021) have used the attribute framework, and they used it as an analytic tool for coding semi-structured interviews about participants’ understanding of systems thinking and their reflections of the strengths and weaknesses of the Australian Healthy Together Victoria prevention system.

A recent scoping review explored the various complex systems methods used in PPAP research as well as the extent to which systems were described in terms of attributes in the framework (Baugh Littlejohns *et al*., 2023). While many attributes and associated dimensions were discussed in the literature, a few studies explicitly explored them to describe a (whole) system for PPAP. Thus, there are ample calls for whole-of-system approaches, and the attribute framework provides one way to identify system boundaries for taking such an approach.

Plsek and Greenhalgh (2001) state that ‘mechanical systems boundaries are fixed and well defined; for example, knowing what is and is not a part of a car is no problem. Complex systems typically have fuzzy boundaries’. Using the above-noted attributes and dimensions as boundaries presents a promising approach. This article reports on using the attribute framework as a way to address the often elusive boundaries in systems research and help describe a complex system for PPAP. The article reports on the first phase of a project to strengthen a system for PPAP within the provincial geographical and administrative structures of British Columbia, Canada. The future second phase of the project will translate key findings of descriptions of the current system into variables for use in participatory system mapping. The overarching aim of the project is to provide evidence-based policy and practice support to stakeholders in the formal Population and Public Health sector (i.e. part of the publicly funded and administered provincial government health care system) to strengthen multisector coordination of PPAP.

## METHODS

Interviews (March–April 2022) and workshops (November–December 2022) were conducted to describe the current system for PPAP in British Columbia from the perspective of key leaders or members of various networks that had a proximal role in PPAP. Ethics approval was received through the Behavioural Research Ethics Board of the University of British Columbia (H21-03328).

### Participants

A list of 78 stakeholders, leaders or members of various networks was created by the project team for recruitment purposes. For interviews, a purposeful sampling method was employed to ensure that there was representation across the province, sectors and levels (provincial, regional and local). Twenty-one potential interviewees were sent an email invitation (and an information letter) to participate in a 1-h online interview and 19 consented interviews (Table 1). For the workshops, all 78 stakeholders were sent an email invitation to participate in one of two scheduled online workshops to boost participation. Participants were offered a $25 electronic gift card as appreciation. Sixteen people participated in the two workshops (Table 1). There were 35 participants in both interviews and the two workshops (Table 1).

**Table 1:** : Number (absolute count) and sector of stakeholders in interviews and workshops

Sector		Interviews	Workshop 1	Workshop 2	Absolute count
Public, provincial government	Ministry of Health	2	1	2	5
	Provincial Health Services (specialized programmes/services)	2	0	0	2
	Ministry of Education	1	0	0	1
	Ministry of Tourism, Art and Culture	1	0	0	1
	Ministry of Transportation and Infrastructure	1	0	0	1
Public, post-secondary education	Medicine/Health Sciences	2	0	0	2
	Education	0	1	0	1
Public, regional	Regional Health Authority, Population and Public Health	5	1	4	10
Public, local	Public, Local/Regional Government	0	0	2	2
Non-profit, provincial	Non-profit, Provincial Organization, Health	3	1	1	5
	Non-profit, Provincial Organization, Sport and or Recreation	2	1	2	5
	Total	19	5	11	35^a^

^a^Two interviewees participated in Workshop 1 and one interviewee participated in Workshop 2.

### Data collection and analysis: interviews and workshops

For the interviews, a semi-structured interview guide was used to explicitly ask questions about attributes in the framework, rate the extent to which attributes were present and explain the rating (available upon request). Ratings (i.e. to little, to some, to great extent) were intended to help focus qualitative descriptions and not for quantitative analysis. As described above, the attributes and dimensions provided boundaries to describe the current system; however, openness to new attributes, dimensions and configurations was desired. All interviews were conducted and audio recorded via Zoom and then transcribed using otter.ai (https://otter.ai/). Audio recorded verbal consent was obtained at the beginning of each interview. All transcriptions were reviewed against the audio recording for accuracy and then saved to NVivo software. Transcripts were coded using directed content analysis (Hsieh and Shannon, 2005) to generate themes regarding each attribute. Interviews were conducted and analysed by L.B.L.; however, project team members (G.M., D.R. and G.F.) were assigned interview transcripts to review and discuss. A *Report on Interviews* summarizing findings by attribute and dimension was prepared as a working document and disseminated with invitations to participate in a workshop (available upon request).

Workshop participants joined a 2.5-h Zoom meeting and used a Google Doc in small groups to report important stories about helping and hindering factors in the current system for PPAP. Following this, using a Miro Board, participants were asked to discuss and identify attributes that they could discern in the stories and create collective diagrams (i.e. connection circles, mind maps) of the interdependence or the connections among attributes and dimensions. A key aim of conducting workshops was to bring stakeholders together to share, hear and engage in dialogue. Stories were seen as a compliment to the deductive approach taken in the interviews, and a way to triangulate data and provide more perspectives of the current system. Thirty-four stories were documented in the two workshops. Data analysis involved saving stories to NVivo software, clustering and coding themes (Patton, 1990) and exploring links among attributes. The linkages and maps were not analysed further, as they were intended to be exploratory tools to enhance systems thinking among participants and project team members. A *Report on Workshops* summarizing the stories and maps was prepared as another working document (available upon request).

### Data synthesis: key findings

Finally, a data synthesis process was adapted from Baugh Littlejohns *et al*. (2018) to report key findings. This included three steps: (i) saving the *Report on Interviews* and *Report on Workshops* to NVivo software, (ii) coding findings by thematically clustering ideas with respect to attributes and dimensions and (iii) developing a list of statements to describe the current system.

## RESULTS

### Adapted attribute framework

**Fig. 2:**
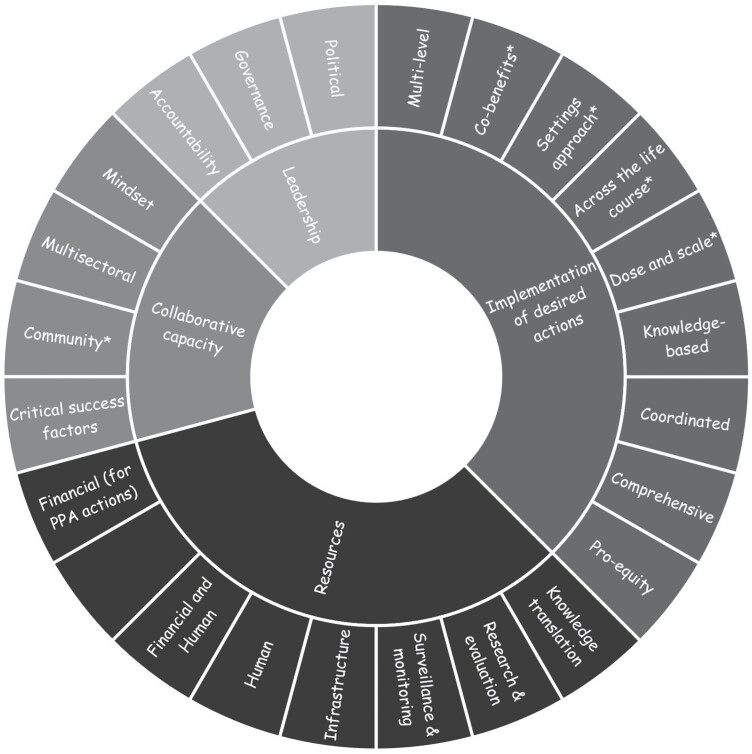
Adapted attribute framework. *New dimensions.

An adapted attribute framework is a notable result of interviews. Many interviewees were clear about how to condense the number of attributes; therefore, a new configuration or a unique framework evolved based on these perspectives (Figure 2). Four attributes (from the original seven) and six new dimensions make up the adapted framework. First, most interviewees conceptualized *Information* (and associated dimensions of surveillance and monitoring, research and evaluation, and knowledge translation) as a dimension of *Resources* and not an attribute unto itself. Second, *Implementation of Desired Actions* now subsumes the attribute of *Health Equity Paradigm*, and from this, *Pro-equity* is a new dimension. Interviewees mostly discussed reducing health equities as part of planning and implementing actions to address physical inactivity. Third, *Complex System Paradigm* was taken out of the framework because interviewees reported that they were in the preliminary phases of learning about integrating complex systems thinking in their research, policy and/or practice. Fourth, regarding other new dimensions, the *Implementation of Desired Actions* attribute now includes *Integrating co-benefits* (e.g. mental health, climate change), using a *Settings approach*, addressing actions across the *Life course* and ensuring appropriate *Dose and scale*. Finally, *Collaborative Capacity* includes a separate *Community* dimension as this was considered essential and distinct from the *Multisectoral* dimension. The adapted framework was used in the analysis and reporting on workshops.

### Descriptions of a provincial system for PPAP

**Fig. 3:**
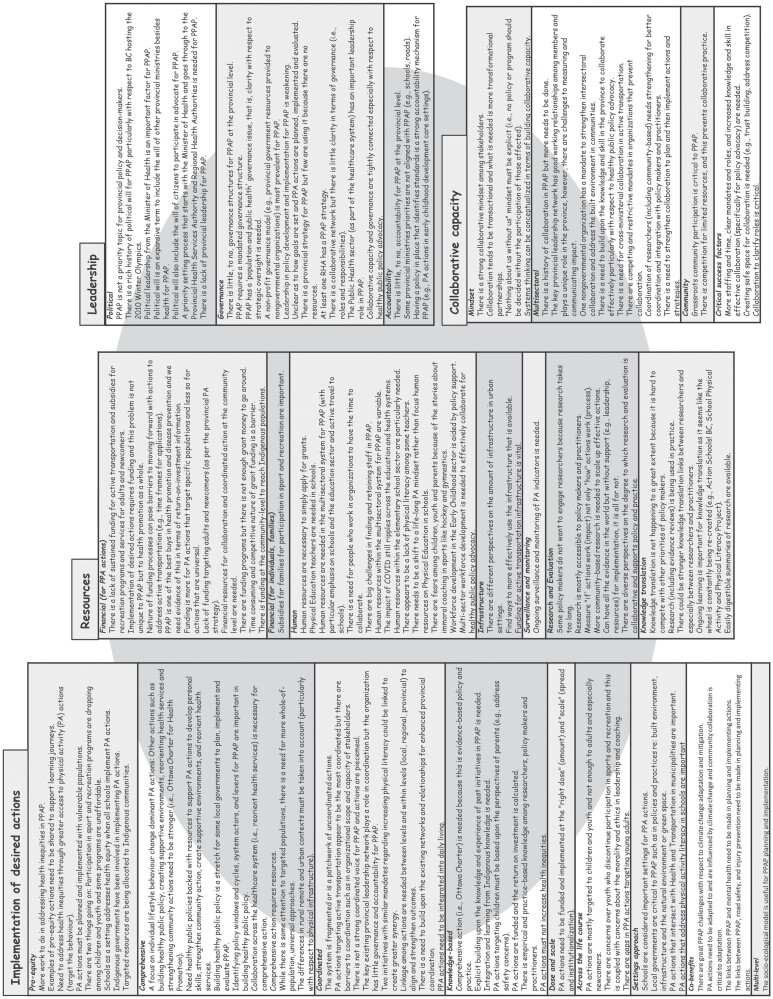
Descriptive statements regarding the system for population physical activity promotion in British Columbia (2022). PPAP refers to population physical activity promotion, PA refers to physical activity. PPA refers to population physical activity and RHA refers to regional health authority.

Based on the adapted framework, 121 statements were developed and organized in terms of attributes and dimensions describing strengths and challenges in the current system for PPAP (Figure 3). The greatest number of statements relate to *Implementation of Desired Actions* (*n* = 44) and closely followed by *Resources* (*n* = 39). *Leadership* (*n* = 21) and *Collaborative Capacity* (*n* = 17) have a lower number of statements.

## DISCUSSION

Nau *et al*. (2022) report that the ‘use of system approaches to increase PA [physical activity] in populations is at a relatively early stage of development, with a preponderance of descriptive approaches and a dearth of more advanced forms of practice and analysis’. They call for further development, implementation and evaluation of systems and complexity-informed approaches. The application of the attribute framework appears to be useful in exploring such approaches. It describes the parts of a system for PPAP in order to help see the whole. Seeing the system through the lens of the attributes and dimensions offers a way to address the challenge of identifying often elusive boundaries in whole-of-system approaches to health promotion and chronic disease prevention. Overall, the 121 statements (Figure 3) provide a rich description of what this unique system ‘does’. The key to seeing the whole, however, is to study the interdependence among the parts because ‘a system is never the sum of its parts it’s the product of their interaction’ (Ackoff, 2019). This is a key challenge for the next phase of the project, that is, to apply the key findings in system mapping and specifically in group model building where causal loop diagrams are co-created. Causal loop diagrams can be used as an analytical tool to study interactions, by embedding system dynamics and feedback theory, and ultimately identify leverage points for systems change (Baugh Littlejohns *et al*., 2021).

There are examples of other frameworks to describe the boundaries of systems for PPAP in the literature. Murphy *et al*. (2021) used the GAPPA action areas to assess the Irish context and found that there is a need to strengthen active systems to support active societies, environments and people. GAPPA action areas for active systems (leadership, governance and policy frameworks, information, research and evaluation, advocacy and financing mechanisms) clearly overlap with attributes and dimensions. In another study, Bellew *et al.* (2020) suggest that a focus on governance, advocacy and knowledge translation mechanisms could strengthen Australian systems for PPAP. These focus areas also overlap with the attributes in the framework. Future implications could include comparing and contrasting GAPPA action areas (including active systems, societies, environments and people) and other explicit whole-of-system focus areas with attributes and dimensions to create an enhanced and integrated framework that represents a desired depth and breadth of system boundaries.

Further to the potential integration of frameworks, defining boundaries of (whole) systems can be conceptualized in terms of critiquing the meaning and the validity of propositions or judgements (Ulrich, 2002). The meaning and validity of boundary judgements ‘inherently privilege what are considered relevant facts (observation) and norms (valuation standards)’ (Ulrich, 2002). The made-in-BC adapted attribute framework represents boundary judgements based upon an initial literature review and developed from enriched meaning found through stakeholder participation (Figure 1). Future implications point to the importance of interrogating theoretical, methodological and empirical propositions in the potential integration of frameworks.

The importance of engaging with diverse system stakeholders is a key tenet of complex systems thinking as well as health promotion and chronic disease prevention (World Health Organization, 1986; Pescud *et al*., 2021; Wenger-Trayner and Wenger-Trayner, 2021; Poon *et al*., 2022). Future implications of applying the attribute framework are necessarily context dependent or specific. Applying the attribute framework in other contexts (e.g. other jurisdictions) and settings (e.g. school systems for PPAP), to specific topics (e.g. built environments) and core research problems (e.g. multisectoral collaboration for PPAP), and comparing and contrasting emergent adapted frameworks, to describe systems for PPAP, would be ideal. This would clearly highlight diversity in the meaning and validity of boundary judgements. Further to this, applying the framework to describe other systems for health promotion and chronic disease prevention (e.g. food security) could be explored.

In terms of methods used, interviews and workshops were useful to gain descriptions of what is happening in the system through the lens of attributes and dimensions. The integration of these methods enriched and expanded the scope of key findings. There are many examples of interviews being used as a first step in complex systems methods in public health research (Eker *et al*., 2018; Owen *et al*., 2018; Jalali *et al*., 2019; Urwannachotima *et al*., 2019; Crielaard *et al*., 2020; Bensberg *et al*., 2021; Burrell *et al*., 2021; Clarke *et al*., 2021; Parmar *et al*., 2021). Interviews have been used to define boundaries or the important variables in systems for PPAP and often in conjunction with literature reviews (Guariguata *et al*., 2021). There are also examples of workshops or group model-building sessions as an approach to studying systems for PPAP, and these too were often used in conjunction with other methods. Specifically, workshops have been used to define system boundaries or variables of interest in PPAP in order to complete causal loop diagrams (Brennan *et al*., 2015; Keane *et al*., 2015; Guariguata *et al*., 2021; Waterlander *et al*., 2021). This article adds a new perspective to this work in that the attribute framework provides a scaffolding for the application of future complex system methods.

Other methodological implications include the potential use of a survey tool to collect descriptive data from stakeholders as opposed to time-intensive interviews and workshops. However, the richness of the descriptions could be lost, and furthermore, workshops are critical to providing a forum for shared sense-making of the system. However, to address these limitations, there are survey tools and software designed to use with participatory processes such as Kurtz’s (2014) participatory narrative inquiry that could be used.

An immediate implication for the project is to translate the list of statements in Figure 2 into variables and facilitate a system mapping workshop or group model-building session to co-create a causal loop diagram in the next phase. The aim of the final phase of the project is to identify leverage points or places to intervene in the system for PPAP (Meadows, 1999). Other methods that may be promising in using descriptive statements, such as in this article, include connection circles (Molloy, nd), participatory systemic inquiry (Morgan *et al*., 2023), conceptual maps (Bellew *et al*., 2020), rich pictures (Conte and Davidson, 2020) and concept maps (Wutzke *et al*., 2017).

### Strengths and limitations

A strength of this study is that, to our knowledge, it is the first time that the full attribute framework has been used in data collection, analysis and reporting to describe a system for PPAP or health promotion. Although we were particularly cognizant of a potential blinkering effect when using the attribute framework, this may be a potential limitation, and we welcome commentary on this. Blinkering is used here to mean the potential for biased adherence to the attributes and dimensions in the framework and/or to not create space for other topics, themes or perspectives to ascend in importance. For example, power, politics and ideology are important characteristics of systems for health promotion (Baugh Littlejohns *et al*., 2018); however, they are not explicit in the attribute framework nor did they surface in the key findings and descriptive statements.

Although there was diverse representation among stakeholders engaged in interviews and workshops, there were perspectives missing. These included the explicit perspectives of specific population groups such as newcomers to Canada; those with racial, ethnic and cultural identities; sex, gender and sexual identities; and age and ability. Despite this, we acknowledge that systems cannot ever be fully described and any model will ‘fall far short of representing the real world fully’ (Meadows, 2008).

The key findings provide rich descriptions of what was currently happening across the system as a whole for PPAP but represent only a snapshot in time. Senge (2006) highlights that thinking in systems is about seeing relationships and patterns as opposed to static snapshots. This article reports on snapshots of attributes and dimensions, and due to time constraints, interviewees and workshop participants were not engaged in data analysis and interpretation of these results. In future applications of these methods, it would be valuable to design participation from the start (i.e. data collection, analysis and reporting) to not only build understanding of attributes and dimensions but also foster preliminary insights into relationships and patterns among them. The next phase of the project will employ participatory system mapping methods to study relationships and patterns and identify leverage points to strengthen the system for PPAP in BC.

## CONCLUSION

The attribute framework was a useful tool to take a whole-of-system approach and turn elusive boundaries into clear descriptors of a provincial system for PPAP. Implications of this research include translating key findings into variables for use in participatory system mapping in BC. Future applications of the attribute framework may be promising in other jurisdictions, contexts and settings, and in studying other health promotion topics, thereby offering potential opportunities to compare and contrast emergent frameworks and results.

## References

[CIT0001] Ackoff, R. (2019) *Ackoff on Systems Thinking*. The Deming Institute. https://deming.org/ackoff-on-systems-thinking-and-management/.

[CIT0002] Baugh Littlejohns, L., Baum, F., Lawless, A. and Freeman, T. (2018) The value of a causal loop diagram in exploring the complex interplay of factors that influence health promotion in a multisectoral health system in Australia. Health Research Policy and Systems, 16, 126. 30594203 10.1186/s12961-018-0394-x PMC6310960

[CIT0003] Baugh Littlejohns, L., Hill, C. and Neudorf, C. (2021) Diverse approaches to creating and using causal loop diagrams in public health research: recommendations from a scoping review. Public Health Reviews, 42, 1604352. 35140995 10.3389/phrs.2021.1604352 PMC8712315

[CIT0004] Baugh Littlejohns, L., Near, E., McKee, G., Rasali, D., Naiman, D. and Faulkner, G. (2023) A scoping review of complex systems methods used in population physical activity research: do they align with attributes of a whole system approach? Health Research Policy and Systems, 21, 18. 36864409 10.1186/s12961-023-00961-3 PMC9979563

[CIT0005] Baugh Littlejohns, L. and Wilson, A. (2019) Strengthening complex systems for chronic disease prevention: a systematic review. BMC Public Health, 19, 729. 31185993 10.1186/s12889-019-7021-9 PMC6558784

[CIT0006] Beer, S. (2002) What is cybernetics? Kybernetes, 31, 209–219.

[CIT0007] Bellew, W., Smith, B. J., Nau, T., Lee, K., Reece, L. and Bauman, A. (2020) Whole of systems approaches to physical activity policy and practice in Australia: the ASAPa project overview and initial systems map. Journal of Physical Activity & Health, 17, 68–73. 31756721 10.1123/jpah.2019-0121

[CIT0008] Bensberg, M., Joyce, A. and Wilson, E. (2021) Building a prevention system: infrastructure to strengthen health promotion outcomes. International Journal of Environmental Research and Public Health, 18, 1618. 33567719 10.3390/ijerph18041618 PMC7914461

[CIT0009] Brennan, L. K., Sabounchi, N. S., Kemner, A. L. and Hovmand, P. (2015) Systems thinking in 49 communities related to healthy eating, active living, and childhood obesity. Journal of Public Health Management and Practice, 21, S55–S69. 10.1097/PHH.0000000000000248 25828223

[CIT0010] Burrell, M., White, A. M., Frerichs, L., Funchess, M., Cerulli, C., DiGiovanni, L. et al. (2021) Depicting ‘the system’: how structural racism and disenfranchisement in the United States can cause dynamics in community violence among males in urban black communities. Social Science & Medicine, 272, 113469. 33601249 10.1016/j.socscimed.2020.113469

[CIT0011] Cavill, N., Richardson, D., Faghy, M., Bussell, C. and Rutter, H. (2020) Using system mapping to help plan and implement city-wide action to promote physical activity. Journal of Public Health Research, 9, 1759. 32913833 10.4081/jphr.2020.1759 PMC7459760

[CIT0012] Clarke, B., Kwon, J., Swinburn, B. and Sacks, G. (2021) Understanding the dynamics of obesity prevention policy decision-making using a systems perspective: a case study of Healthy Together Victoria. PLoS One, 16, e0245535. 33481898 10.1371/journal.pone.0245535 PMC7822316

[CIT0013] Conte, K. P. and Davidson, S. (2020) Using a ‘rich picture’ to facilitate systems thinking in research coproduction. Health Research Policy and Systems, 18, 14. 32005252 10.1186/s12961-019-0514-2 PMC6995183

[CIT0014] Crielaard, L., Dutta, P., Quax, R., Nicolaou, M., Merabet, N., Stronks, K. et al. (2020) Social norms and obesity prevalence: from cohort to system dynamics models. Obesity Reviews, 21, e13044. 32400030 10.1111/obr.13044 PMC7507199

[CIT0015] Eker, S., Zimmermann, N., Carnohan, S. and Davies, M. (2018) Participatory system dynamics modelling for housing, energy and wellbeing interactions. Building Research & Information, 46, 738–754.

[CIT0016] Guariguata, L., Unwin, N., Garcia, L., Woodcock, J., Samuels, T. A. and Guell, C. (2021) Systems science for developing policy to improve physical activity, the Caribbean. Bulletin of the World Health Organization, 99, 722–729. 34621090 10.2471/BLT.20.285297 PMC8477427

[CIT0017] Hawe, P., Shiell, A. and Riley, T. (2009) Theorising interventions as events in systems. American Journal of Community Psychology, 43, 267–276. 19390961 10.1007/s10464-009-9229-9

[CIT0018] Holdsworth, M., Nicolaou, M., Langoien, L. J., Osei-Kwasi, H. A., Chastin, S. F. M., Stok, F. M. et al. (2017) Developing a systems-based framework of the factors influencing dietary and physical activity behaviours in ethnic minority populations living in Europe—a DEDIPAC study. The International Journal of Behavioral Nutrition and Physical Activity, 14, 154. 29115995 10.1186/s12966-017-0608-6 PMC5678802

[CIT0019] Hsieh, H.-F. and Shannon, S. E. (2005) Three approaches to qualitative content analysis. Qualitative Health Research, 15, 1277–1288. 16204405 10.1177/1049732305276687

[CIT0020] Jalali, M. S., Rahmandad, H., Bullock, S. L., Lee-Kwan, S. H., Gittelsohn, J. and Ammerman, A. (2019) Dynamics of intervention adoption, implementation, and maintenance inside organizations: the case of an obesity prevention initiative. Social Science & Medicine (1982), 224, 67–76. 30763824 10.1016/j.socscimed.2018.12.021 PMC8167908

[CIT0021] Keane, P., Ortega, A. and Linville, J. (2015) Healthy kids, healthy Cuba: findings from a group model building process in the rural Southwest. Journal of Public Health Management and Practice, 21 (Suppl 3), S70–S73. 25828224 10.1097/PHH.0000000000000250

[CIT0022] Krueger, H., Rasali, D. and Fong, D. (2018) *The Economic Burden of Risk Factors in British Columbia, 2015: Excess Weight, Tobacco Smoking, Alcohol Use, Physical Inactivity and Low Fruit and Vegetable Consumption*. http://www.bccdc.ca/pop-public-health/Documents/economic_burden_five_risk_factors_BC_2015.pdf (15 January 2022, date last accessed).

[CIT0023] Kurtz, C. (2014) Working With Stories in Your Community or Organization. Kurtz-Fernhout Publishing, Albany, New York, USA.

[CIT0024] Meadows, D. (1999) *Leverage Points: Places to Intervene in a System*. https://donellameadows.org/wp-content/userfiles/Leverage_Points.pdf (15 January 2022, date last accessed).

[CIT0025] Meadows, D. (2008) Thinking in Systems: A Primer. Chelsea Green Publishing Co, White River Junction, VT.

[CIT0026] Molloy, J. (nd) *Learning About Connection Circles*. https://thesystemsthinker.com/learning-about-connection-circles/ (15 January 2022, date last accessed).

[CIT0027] Morgan, M. J., Stratford, E., Harpur, S. and Rowbotham, S. (2023) A systems thinking approach for community health and wellbeing. Systemic Practice and Action Research. 10.1007/s11213-023-09644-0

[CIT0028] Murphy, J. J., Mansergh, F., Murphy, M. H., Murphy, N., Cullen, B., O’Brien, S. et al. (2021) ‘Getting Ireland active’—application of a systems approach to increase physical activity in Ireland using the GAPPA framework. Journal of Physical Activity and Health, 18(11), 1–10. 10.1123/jpah.2020-0864 34583322

[CIT0029] Nau, T., Bauman, A., Smith, B. J. and Bellew, W. (2022) A scoping review of systems approaches for increasing physical activity in populations. Health Research Policy and Systems, 20, 104. 36175916 10.1186/s12961-022-00906-2 PMC9524093

[CIT0030] Owen, B., Brown, A. D., Kuhlberg, J., Millar, L., Nichols, M., Economos, C. et al. (2018) Understanding a successful obesity prevention initiative in children under 5 from a systems perspective. PLoS One, 13, e0195141. 29596488 10.1371/journal.pone.0195141 PMC5875853

[CIT0031] Parmar, P. K., Rawashdah, F., Al-Ali, N., Rub, R. A. A., Fawad, M., Amire, K. A. et al. (2021) Integrating community health volunteers into non-communicable disease management among Syrian refugees in Jordan: a causal loop analysis. BMJ Open, 11, e045455. 10.1136/bmjopen-2020-045455 PMC8061821 33879489

[CIT0032] Patton, M. Q. (1990) Qualitative Evaluation and Research Methods. SAGE Publications, Los Angeles, California, USA.

[CIT0033] Pescud, M., Rychetnik, L., Allender, S., Irving, M. J., Finegood, D. T., Riley, T. et al. (2021) From understanding to impactful action: systems thinking for systems change in chronic disease prevention research. Systems, 9, 61. https://www.mdpi.com/2079-8954/9/3/61

[CIT0034] Plsek, P. and Greenhalgh, T. (2001) The challenge of complexity in health care. BMJ Open, 323, 625–628. 10.1136/bmj.323.7313.625 PMC1121189 11557716

[CIT0035] Poon, B. T., Atchison, C., Kwan, A. and Veasey, C. (2022) A community-based systems dynamics approach for understanding determinants of children’s social and emotional well-being. Health & Place, 73, 102712. 34808588 10.1016/j.healthplace.2021.102712

[CIT0036] Rutter, H., Cavill, N., Bauman, A. and Bull, F. (2019) Systems approaches to global and national physical activity plans. Bulletin of the World Health Organization, 97, 162–165. 30728623 10.2471/BLT.18.220533 PMC6357559

[CIT0037] Santos, A. C., Willumsen, J., Meheus, F., Ilbawi, A. and Bull, F. C. (2023) The cost of inaction on physical inactivity to public health-care systems: a population-attributable fraction analysis. The Lancet Global Health, 11, e32–e39. 36480931 10.1016/S2214-109X(22)00464-8 PMC9748301

[CIT0038] Senge, P. (2006) The Fifth Discipline. Currency Books, New York, USA.

[CIT0039] Ulrich, W. (2002) Boundary critique. In Daellenbach, H. and Flood, R. (eds), The Informed Student Guide to Management Science. Thomson Learning, Boston, MA, USA.

[CIT0040] Urwannachotima, N., Hanvoravongchai, P. and Ansah, J. P. (2019) Sugar-sweetened beverage tax and potential impact on dental caries in Thai adults: an evaluation using the group model building approach. Systems Research and Behavioral Science, 36, 87–99.

[CIT0041] Waterlander, W. E., Singh, A., Altenburg, T., Dijkstra, C., Luna Pinzon, A., Anselma, M. et al. (2021) Understanding obesity-related behaviors in youth from a systems dynamics perspective: the use of causal loop diagrams. Obesity Reviews, 22, e13185. 33369045 10.1111/obr.13185 PMC8243923

[CIT0042] Wenger-Trayner, E. and Wenger-Trayner, B. (2021) *Systems Convening: A Crucial Form of Leadership for the 21st Century*. https://wenger-trayner.com/systems-convening/.

[CIT0043] World Health Organization. (1986) *Ottawa Charter for Health Promotion*. https://www.who.int/teams/health-promotion/enhanced-wellbeing/first-global-conference.

[CIT0044] World Health Organization. (2018) *Global Action Plan on Physical Activity 2018–2030: More Active People for a Healthier World*. https://www.who.int/publications/i/item/9789241514187 (15 January 2022, date last accessed).

[CIT0045] World Health Organization. (2022) *Global Status Report on Physical Activity 2022*. https://www.who.int/publications/i/item/9789240059153 (15 January 2022, date last accessed).

[CIT0046] Wutzke, S., Roberts, N., Willis, C., Best, A., Wilson, A. and Trochim, W. (2017) Setting strategy for system change: using concept mapping to prioritise national action for chronic disease prevention. Health Research Policy and Systems, 15, 69. 28784177 10.1186/s12961-017-0231-7 PMC5547536

